# Supplementary material for: The cuttlefish Sepia officinalis (Sepiidae, Cephalopoda) constructs cuttlebone from a liquid-crystal precursor
Source: Sci Rep. 2015 Jun 18;5:11513. doi: 10.1038/srep11513 (PMC4471886; doi:10.1038/srep11513)
Supplement: Supplementary Information [file srep11513-s1.pdf]

The cuttlefish *Sepia officinalis* (Sepiidae, Cephalopoda) constructs cuttlebone from a liquid-crystal precursor

Antonio G. Checa[1*], Julyan H.E. Cartwright[2], Isabel Sánchez-Almazo[3], José P. Andrade[4], Francisco Ruiz-Raya[5]

[1]Departamento de Estratigrafía y Paleontología, Universidad de Granada, 18071 Granada, Spain. [2]Instituto Andaluz de Ciencias de la Tierra, CSIC-Universidad de Granada, 18071 Granada, Spain. [3]Centro de Instrumentación Científica, Universidad de Granada, 18071 Granada, Spain. [4]Centro de Ciências do Mar do Algarve, Universidade do Algarve, Faro, Portugal. [5]Departamento de Zoología, Universidad de Granada, 18071 Granada, Spain.

*e-mail: acheca@ugr.es

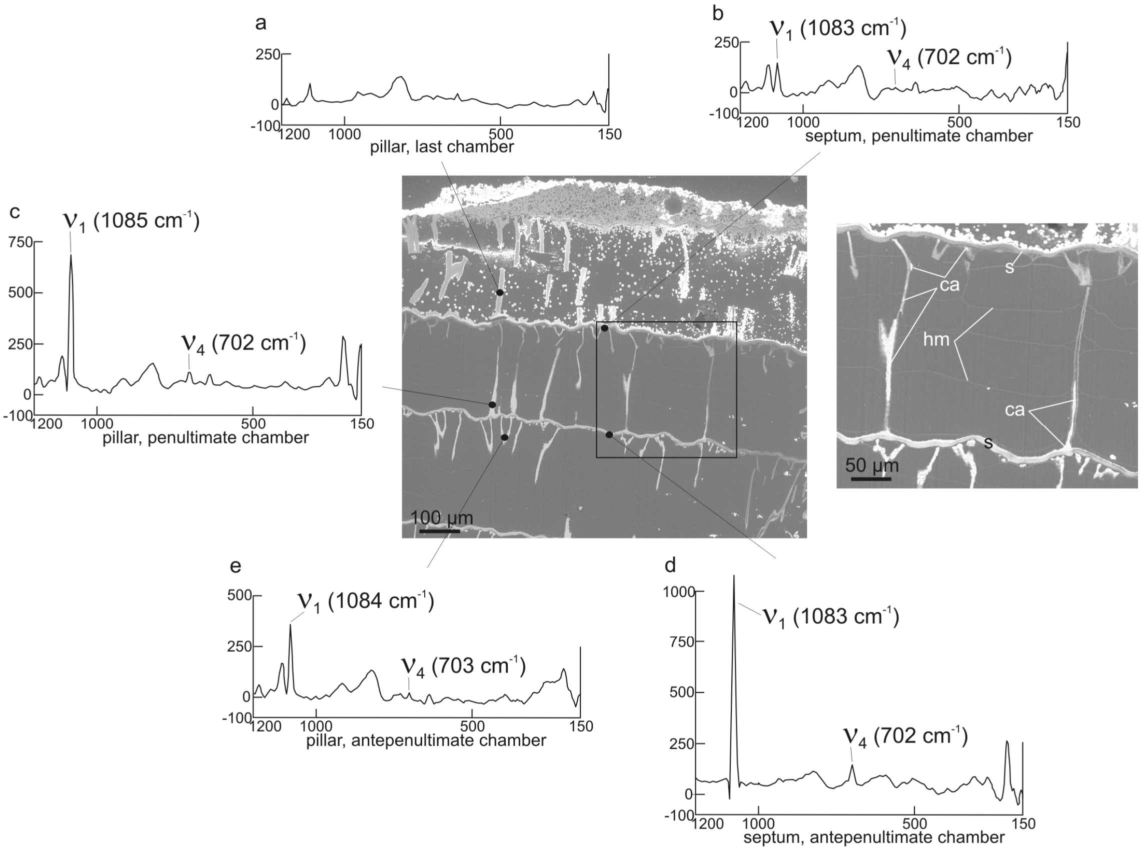

Figure S1 | Raman analysis of the last formed chambers of an embedded juvenile cuttlebone. When present, the most intense ν_1 and ν_4 bands of aragonite are indicated. According to the spectra, the last chamber (**a**) is totally unmineralized, the penultimate septum (**b**) is slightly mineralized, and the pillars of the penultimate chamber (**c**) and all the elements of the antepenultimate chamber are mineralized with aragonite (**d**, **e**). The right image is a close up of the framed area, where it can be appreciated that only the bases and some additional spots of the pillars, as well as the penultimate septum, are mineralized. The SEM images have been acquired in back scattered mode. ca= calcified areas, hm= horizontal membranes, s= septa.

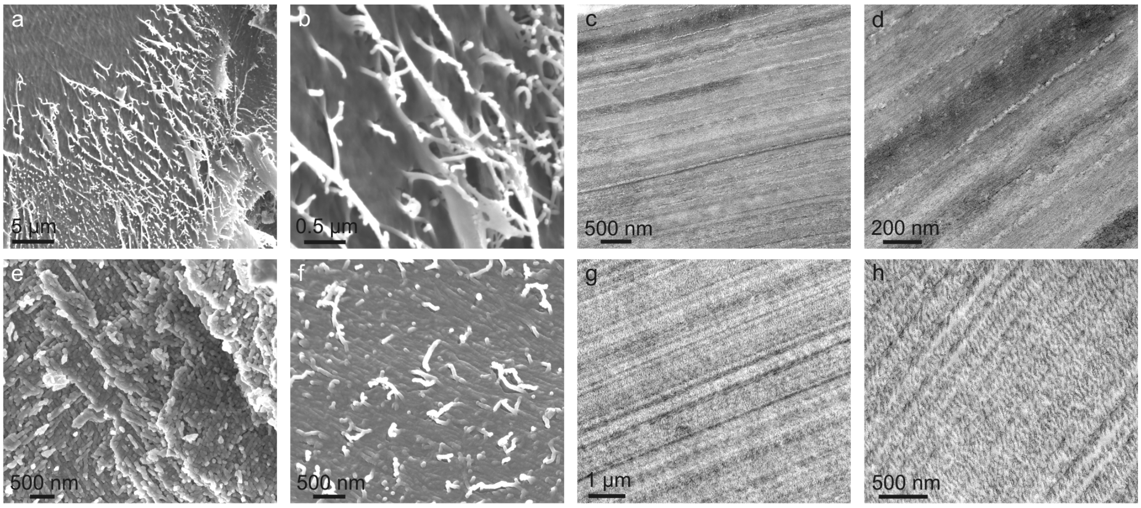

Figure S2 | Structures of the dorsal shield of the cuttlebone of *Sepia* and of the septum of *Spirula*. **a**-**d**, Dorsal shield of the cuttlebone of *Sepia officinalis*. **a**, SEM view showing the layered aspect. Each layer is made of co-oriented fibres ~50 nm thick. **b**, Close-up view of **a**. **c**, **d**, TEM views; the layered aspect is evident. **e**-**h**, Septum of *Spirula spirula* (Linnaeus, 1758). **e**, Oblique fracture showing the microstructure composed of layers of co-oriented fibres of aragonite (70-90 nm thick) at high angles to those of adjacent planes. **f**, Surface view of the decalcified septum showing the arrangement of co-oriented organic fibres. **g**, TEM view showing the layered aspect and the arcuate internal distribution of some of the layers. **h**, Detail of **g**, in which the individual fibres of, presumably, chitin, can be discerned; they have thicknesses between 10 and 20 nm.

Supplementary Video S1 | Tomographic reconstruction of a fragment of the last three chambers of a subadult specimen of *Sepia officinalis*. The cut out area of the last chamber is the siphuncular area. Only the calcified elements are shown. The video is intended to show (1) the lack of connection of the pillars of the last chamber with the chamber roof, due to incomplete calcification, (2) the peculiar, antler-like morphology of pillars of the siphuncular area and (3) the anteroposterior alignment of the pillars, which is particularly noticeable at their dorsal ends. The venter is to the top.
